# SOMATIC COMORBIDITY PATTERNS IN PEOPLE WITH DEPRESSION: A POPULATION-BASED COHORT STUDY

**DOI:** 10.1093/geroni/igad104.0675

**Published:** 2023-12-21

**Authors:** Hao Luo, Davide Vetrano, Federico Triolo, Alberto Zucchelli, Yingyang Zhang, Pengcheng Wang, Kenneth Man, Francisco Lai

**Affiliations:** The University of Hong Kong, Hong Kong, Hong Kong; Karolinska Institutet, Solna, Stockholms Lan, Sweden; Aging Research Center, Solna, Stockholms Lan, Sweden; Università degli Studi di Brescia, Brescia, Lombardia, Italy; The University of Hong Kong, Hong Kong, Hong Kong; HKU, Hong Kong, Hong Kong; University College London, London, England, United Kingdom; The University of Hong Kong, Hong Kong, Hong Kong

## Abstract

People with depression often present with co-existing somatic conditions, which impact survival and steer patients’ clinical management. This study aimed to identify unique patterns of somatic comorbidity in people with depression and examine their associated mortality. We used population-based electronic health records from the Clinical Data Analysis and Reporting System in Hong Kong. People aged 40+ who had an incident depression diagnosis between 2001 and 2015 were included and followed up until 31 December 2018. Latent class analysis was applied, separately by decades, to individuals with depression and at least one comorbid somatic condition. Cox proportional hazard models were used to examine the risk of mortality associated with specific comorbidity patterns, adjusting for sociodemographic and clinical characteristics. We identified 38,889 eligible individuals (mean age: 62.8; 68.7% women). Latent class analysis identified an increasing number of patterns with age (two to seven). Compared with the unspecific pattern, the following patterns were associated with the highest mortality risk in different age groups: cardiometabolic and cerebrovascular diseases in individuals aged 40-49 (hazard ratio [HR] 2.5 [95% CI 1.7-3.7]), cardiometabolic diseases and anemia in those aged 50-59 (HR 3.4 [2.6-4.5]) and 70-79 (2.7 [2.3-3.3]), cardiometabolic, cerebrovascular diseases, and anemia in those aged 60-69 (HR 2.5 [2.0-3.2]), and cardiorespiratory disease in those aged 80+ (HR 2.0 [1.8-2.3]). Specific somatic comorbidity patterns were independently and substantially associated with the risk of mortality. This highlights the importance of taking combinations of somatic conditions into account when tailoring the clinical and care approach to people with depression.

